# Detecting TF-miRNA-gene network based modules for 5hmC and 5mC brain samples: a intra- and inter-species case-study between human and rhesus

**DOI:** 10.1186/s12863-017-0574-7

**Published:** 2018-01-22

**Authors:** Ujjwal Maulik, Sagnik Sen, Saurav Mallik, Sanghamitra Bandyopadhyay

**Affiliations:** 1 Department of Computer Science and Engineering, Jadavpur University, Kolkata, 700032 India; 2 Machine Intelligence Unit, Indian Statistical Institute, 203 B.T. Road, Kolkata, 700108 India

**Keywords:** Multiple cytosine variants, Differentially 5hmC-methylated genes, Intra-species co-methylated gene-modules, Differentially multi-stage methylated genes, Inter-species multi-stage co-methylated gene-modules, Weighted TOM and dynamic tree cut, Top regulator transcription factors, Top targeter miRNAs

## Abstract

**Background:**

Study of epigenetics is currently a high-impact research topic. Multi stage methylation is also an area of high-dimensional prospect. In this article, we provide a new study (intra and inter-species study) on brain tissue between human and rhesus on two methylation cytosine variants based data-profiles (viz., 5-hydroxymethylcytosine (5hmC) and 5-methylcytosine (5mC) samples) through TF-miRNA-gene network based module detection.

**Results:**

First of all, we determine differentially 5hmC methylated genes for human as well as rhesus for intra-species analysis, and differentially multi-stage methylated genes for inter-species analysis. Thereafter, we utilize weighted topological overlap matrix (TOM) measure and average linkage clustering consecutively on these genesets for intra- and inter-species study.We identify co-methylated and multi-stage co-methylated gene modules by using dynamic tree cut, for intra-and inter-species cases, respectively. Each module is represented by individual color in the dendrogram. Gene Ontology and KEGG pathway based analysis are then performed to identify biological functionalities of the identified modules. Finally, top ten regulator TFs and targeter miRNAs that are associated with the maximum number of gene modules, are determined for both intra-and inter-species analysis.

**Conclusions:**

The novel TFs and miRNAs obtained from the analysis are: MYST3 and ZNF771 as TFs (for human intra-species analysis), BAZ2B, RCOR3 and ATF1 as TFs (for rhesus intra-species analysis), and mml-miR-768-3p and mml-miR-561 as miRs (for rhesus intra-species analysis); and MYST3 and ZNF771 as miRs(for inter-species study). Furthermore, the genes/TFs/miRNAs that are already found to be liable for several brain-related dreadful diseases as well as rare neglected diseases (e.g., wolf Hirschhorn syndrome, Joubarts Syndrome, Huntington’s disease, Simian Immunodeficiency Virus(SIV) mediated enchaphilits, Parkinsons Disease, Bipolar disorder and Schizophenia etc.) are mentioned.

**Electronic supplementary material:**

The online version of this article (doi:10.1186/s12863-017-0574-7) contains supplementary material, which is available to authorized users.

## Background

DNA methylation placed at the 5th position of cytosine (viz., 5mC or 5-methylcytosine) [1–3] is one of the most important epigenetic factors which plays a significant role in genome regulation, host-defending, and inactivation of X-chromosome ([4–7]). 5-methylcytosine (5mC) is predominantly observed in CpG dinucleotides of the mammalian genome. It is well-known that individual CpGs which are occupied in different parts of the genome, are differentially methylated depending on the category of tissue/cell and development-phase. DNA methylation is catalysed by several enzymes (viz., DNA methyltransferases (DNMTs):- DNMT1, DNMT3A, DNMT3B and DNMT3L) [8, 9]. Furthermore, transcriptional status of gene and the density of GC affect the status of DNA methylation e.g., the maximum parts of CpG islands (symbolized as CGIs) which contain a closely compacted CpG content, are basically hypo-methylated; whereas the remaining portions of the genome including dispersed CpGs in the regions of gene-coding, and the heterochromatin regions, having repetitive CpGs are hyper-methylated in general. Interestingly, it is still under debate that how genome-wide methylation is differentially regulated in the different discrete loci, and how it has been working dynamically in separate cell-categories at the development-stage. However, DNA methylation decreases the gene expression level in general, and therefore plays an crucial task in gene-silencing [1, 2, 10, 11].

Oxidative product of 5mC is 5-hydroxymethylcytosine (5hmC). The 5hmC mark was discovered approximately six decades ago in T-even bacteriophage [12]. Later it has been identified in vertebrate-brain and various other tissues (as mentioned in [13–15]). The 5hmC is found in the embryonic stem cells of mouse at high-level. Although level deteriorates after differentiation of the embryonic stem cells (according to [16, 17]), but increased again in terminally differentiated cells (viz., Purkinje neurons) (according to [13]). Currently, 5hmC is observed in zygotes of bovines, mice and rabbits, and it is collected specifically at paternal pro-nucleus concurring with reduction of 5mC ([18, 19]). The members of several translocation (TET) protein-family mediates the 5hmC. Here, each protein consists of a C-terminal oxidase region. An effective chemical methodology is recently proposed in order to capture and label the 5hmC which shows the map of first distribution for the 5hmC in a brain genome of mouse (i.e., mammal), and the enrichment of it in the highly transcribed genes. Hence, the involvement of 5hmC in the specified gene-bodies during differentiation and maturation of neufirons notifies that the 5hmC is temporally and spatially distributed on brain tissue at the time of the brain development. Conversion of 5mC to 5hmC is responsible for passive DNA methylation. 5hmC is divided in three categories in terms of functions, 5hmC-A, 5hmC-B and 5hmC-C. Among them 5hmC-A are mostly protecting DNA replication whereas 5hmC-C can affect the functional annotations of DNA binding proteins.

Differential methylation is stated to have significant differences in methylation values of a gene across the group of cancer (diseased/experimental) samples and the group of normal (control) samples. Statistical test is a technique of statistical inference which is applied for measuring the differential methylation between two groups (populations). Differential methylation analysis is carried out for determining whether methylation levels of the genes change significantly in terms of mean and/or standard deviation across samples of experimental and control groups. Co-methylation is another important term that basically signifies that the methylation levels of these genes might increase or decrease together in between a sub-range of time-series rather than the overall time-line. Statistical test is important to detect differential methylation samples. Co-methylation provides an insight that differential methylation on one gene sample can become a cause for differential methylation of other gene samples. It can help to understand unusual behavior of certain path having these gene memebers.

Many studies have already been performed on 5-methylcytosine (5mC) for finding disease related information. Recently, researchers have changed their focus from only considering 5mC to including another methylation mark 5-hydroxymethylcytosine (5hmC) with it for observing the variation of regulation due to higher-dimensional methylation marks. Xu et al. (2011) [20] performed an integrated study using genome-wise regulation of 5mC, 5hmC, gene expression by Tet1 proteins in the embryonic stem cells of mouse; whereas Wu et al. (2011) [21] provided another integrated study of 5hmC and gene expression data for wild-type and Tet1-depleted embryonic stem cells of mouse. Tan and Shi (2012) [3] provided information regarding the involvement of 5hmC and Tet proteins in cancer. Bradley et al. (2013) [22] carried out an analysis for determining whether methylation status is changed throughout the progressive stages (phases) of Alzheimer’s disease considering the levels of TET1 protein, 5mC, 5hmC and other intermediate forms of 5hmC. A review by Sun et al. (2014) [23] summarized the latest information regarding the effect and function of 5hmC in the tissue of brain especially focusing on neuron-related actions, and diseases. Al-Mahdawi et al. (2014) [24] performed similar type of survey including the effective role of 5hmC for the neurondegenerative diseases. Chopra et al. (2014) [25] produced an array-dependent assay for determining 5mC and 5hmC in the genomes of several mammals (including human), and represented its generalization to all types of mammals. Condliffe et al. (2014) [26] provided a study that implied epigenetic modifications by different methylation marks (including 5hmC) in Alzheimer’s disease. The aim of all of these is to investigate the functional alternations based on the different cytosine modifications across the samples of two separate anatomic brain regions (viz., cerebellum and entorhinal cortex) of Alzheimer (diseased/experimental) patients and control (normal) patients. However, according to literature survey, gene regulatory network based module detection approach using both 5mC and 5hmC marks has never been applied in the past. Hence, in this article, we provide intra- and inter-species study on brain tissue mostly cerebellum and hippocampal tissue between human and rhesus (monkey) on 5mC and 5hmC data-profiles through TF-miRNA-gene network based module identification to explore the individual and cytosine-variant depended affect on this disease detection.

## Methods

In this section, a proposed framework for intra-species study on multiple cytosine based methylation data (having 5hmC and 5hmC samples) for human as well as rhesus through TF-miRNA-gene network based module identification is presented. For intra-species, we first identify differentially 5hmC-methylated genes, and then compute traditional (weighted) topological overlap measure (TOM) on each pair of the resulting genes. Subsequently, we apply average linkage hierarchical clustering technique using the corresponding dissimilarity values of the TOM scores, and generate a dendrogram of genes. Thereafter, intra-species co-methylated gene-modules are identified from the dendogram, by performing dynamic tree cutter algorithm. An intra-species co-methylated gene-module consists of the genes which are not only correlated in 5mC samples but also in the 5hmC samples for the same category or species (e.g. human or rhesus). Moreover, we carry out literature search as well as Kyoto Encyclopedia of Genes and Genomes (KEGG) pathway and gene-ontology analysis using David database for biological verification of the genes representing the modules for each case. The transcription factors (TFs) that regulate the genes belonging to the different intra-species co-methylated gene-modules, are collected from Transfac and ITFP databases [27]. The miRNAs that target the genes belonging to the different intra-species co-methylated gene-modules, are accumulated from miRTarBase and PITA databases [27]. For the case of inter-species, we initially find differentially multi-stage methylated genes. Thereafter, we apply the aforesaid procedure to intra species as well. An inter-species multi-stage co-methylated gene-module consists of those genes which are correlated in both of 5mC and 5hmC samples of human and rhesus. The transcription factors (TFs) which regulate the genes belonging to the different inter-species co-methylated gene-modules, and the miRNAs which target the genes belonging to the different inter-species co-methylated gene-modules, are determined. For the case of intra-species as well as inter-species, the top ten TFs/miRNAs that link with maximum gene-modules, are determined. Thereafter, among the top ten TFs/miRNAs of both (intra-species or inter-species), we identify the novel (unknown) TFs and miRNAs which have no previous disease-related information in literature. These TFs/miRNAs might be highly liable for any brain related disease. Additionally, the genes/TFs/miRNAs that are already found to be associated with several brain-related dreadful diseases as well as rare neglected diseases (e.g., wolf Hirschhorn syndrome, Joubarts Syndrome, Huntingtons disease, SIV mediated enchaphilits, Parkinsons Disease, Bipolar disorder and Schizophenia etc.) are mentioned.

### Methodology for intra-species analysis

The sets of 5-hydroxymethylcytosine (5hmC) samples and 5-methylcytosine (5mC) samples for the brain tissue for human as well as rhesus are first collected7. For each case of intra-species analyses (i.e., for human as well as rhesus), 5mC samples are considered as controlled samples whereas 5hmC samples are utilized as experimental samples.

### Notification of differentially 5hmC-methylated genes

Since we deal with very small number of samples for intra-species study, therefore simple fold-change method [28] is applied in our study for identifying differentially 5hmC-methylated genes. Fold change for each gene for each intra-species study (viz., *FC*_*intra*_) is defined as the fraction of the mean of the 5hmC samples (experimental group) to the mean of the 5mC samples (control group) for each species.

1 $$ {FC}_{intra}(i)= \frac{\bar{x}_{5hmC}(i)}{\bar{x}_{5hmC}(i)},  $$

where $\bar {x}_{5hmC} (i)$ stands for the mean of the values across 5hmC samples for a gene *i*, and $\bar {x}_{5hmC} (i)$ stands for the mean of the values across 5hmC samples for the gene *i*. Notably, we set a common threshold i.e., 0.25 for both the human and rhesus for the intra-species study.

Thus, for each species (human and rhesus), we find different genesets having the differentially 5hmC-methylated genes. For this case, the collective list of the differentially 5hmC-methylated genes for human is symbolized as $DM^{human}_{5hmC}$ whereas differentially 5hmC-methylated genes for rhesus is denoted as $DM^{rhesus}_{5hmC}$.

### Computing co-methylation using weighted TOM

In order to measure the correlation in terms of methylation levels for intra-species study, we initially perform Pearson’s correlation between pairwise genes belonging to the geneset $DM^{human}_{5hmC}$. Thereafter, we apply weighted topological matrix (http://hms-dbmi.github.io/scw/WGCNA.html) as a weighted connectivity measure for the geneset $DM^{human}_{5hmC}$ where Pearson’s correlation scores are utilized as the weighted-values for adjacency matrix. The mentioned weighted topological matrix score between two vertices *i* and *j* for each intra-species study (i.e., *TOM*_*intra*_(*i*,*j*)) can be stated as follows.

2 $$ {TOM}_{intra}(i,j) =\left\{\begin{array}{lc} \frac{\sum\limits_{v\neq i,j} A(i,v) A(j,v) + A(i,j)}{min\left\{\sum\limits_{v\neq i}A(i,v), \sum\limits_{v\neq j}A(j,v)\right\}- A(i,j)+1}, & \text{if \(i\neq j\)},\\ 1, & \text{if i==j}, \end{array}\right.  $$

where *A* denotes weighted adjacency matrix of the nodes having Pearson’s correlation values as the weights for each intra-species study.

We thereafter compute the corresponding dissimilarity scores of *TOM*_*intra*_ values (similarity scores).

3 $$ {dissim}_{intra}(i,j) = 1 - {TOM}_{intra}(i,j).  $$

Average linkage hierarchical clustering [29] is then utilized on the genes using *dissim*_*intra*_ scores that is represented as:

4 $$ L_{intra}(q,r)=\frac{1}{n_{q} n_{r}} \sum\limits_{i \in C_{q}} \sum\limits_{j \in C_{r}} Da(y_{i}, y_{j}),  $$

where

5 $$ Da(i,j)={dissim}_{intra}(i,j).  $$

Here *q* and *r* are two clusters made for each intra-species, Da(.,.) stands for distance between a point of first cluster and a point of second cluster for each intra-species, and *L*_*intra*_(.,.) denotes the distance between two clusters using average linkage clustering for each intra-species. Then the corresponding dendrogram of the clusters are found.

Dynamic tree cutter [30] is then applied on the dendrogram using color-thresholding method. As a result, intra-species co-methylated gene-modules for human (viz., $GM^{human}_{5hmC}$) are identified. Similarly, we consider the geneset $DM^{rhesus}_{5hmC}$ and follow the same sub-step to produce intra-species co-methylated gene-modules for rhesus (viz., $GM^{rhesus}_{5hmC}$).

### TF-miRNA-gene network analysis and biological verification

**Fig. 1:**
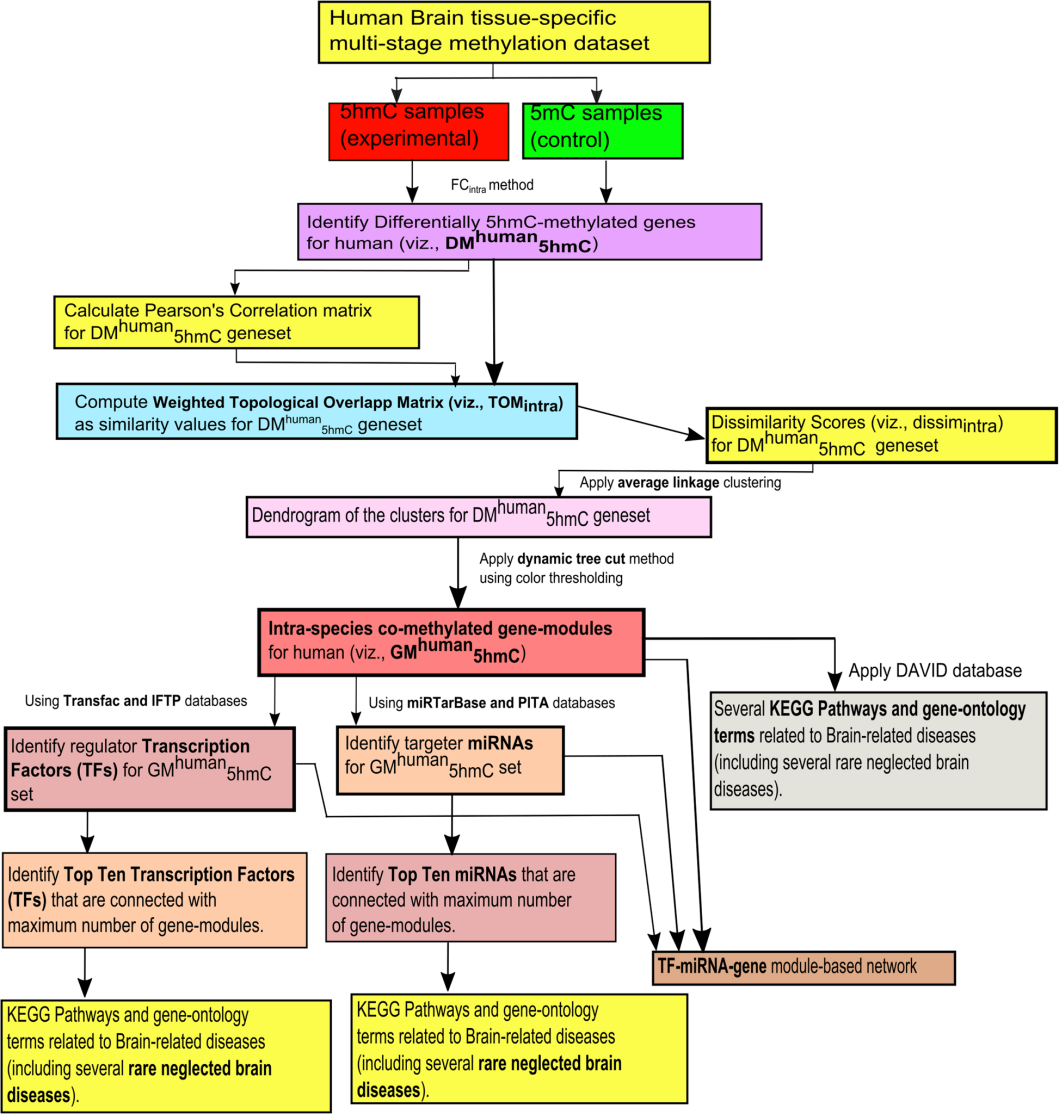
Flowchart of the proposed framework of gene-module detection for human brain-specific multi-stage methylated dataset (i.e., intra-species study for human)

We carry out KEGG pathway and gene-ontology analyses individually using DAVID database on the genes of each module of $GM^{human}_{5hmC}$ in order to verify them. In addition, we collect the transcription factors (TFs) using ITFP and Transfac databases [27] that regulate the genes belonging to the geneset $DM^{human}_{5hmC}$. Similarly, we determine the miRNAs (miRs) using miRTarBase and PITA databases [27] which target the genes of $DM^{human}_{5hmC}$. The top ten regulator TFs that relate to maximum gene-modules, are identified; whereas top ten targeter miRNAs that connect maximum gene-modules, are determined. Notably, when more than one TFs/miRNAs are related to same number of gene-modules, then these TFs/miRNAs are further ranked in descending order of the maximum number of participating regulated/targeted genes from the different modules. Thereafter, the top TFs/miRNAs are verified by literature search in order to know how many of them have a relation with the corresponding diseases related to the sample tissue. The complete pictorial representation of our framework of gene-module detection for the intra-species study for the human is provided in Fig. 1.

In a similar way, KEGG pathway and gene-ontology analyses are performed separately on the genes of each gene-module of $GM^{rhesus}_{5hmC}$ in order to verify them. Besides that, the transcription factors (TFs) which regulate the genes of the geneset $DM^{rhesus}_{5hmC}$, are determined using ITFP and Transfac databases [27]; whereas the miRNAs (miRs) that target the genes belonging to the geneset $DM^{rhesus}_{5hmC}$, are identified through the use of miRTarBase and PITA databases [27]. Thereafter, the top ten regulator TFs which connect with the highest number of gene-modules, are identified; whereas top ten targeter miRNAs which are associated with maximum number of gene-modules, are determined. Notably, when more than one TF/miRNA are related to same number of gene-modules, then these TFs/miRNAs are further ranked in descending order of the maximum number of participating regulated/targeted genes from the different modules. However, the top TFs/miRNAs are thereafter verified by literature search for knowing how many of them have association with the corresponding diseases related to the sample tissue.

### Methodology for inter-species analysis

In this section, we elaborate the proposed framework for inter-species study on multiple cytosine based methylation data (having 5mC and 5hmC samples) between human and rhesus through TF-miRNA-gene network based module identification. The steps of the proposed inter-species framework are described as follows:

In case of inter-species study, we consider both the 5mC and 5hmC samples for the same tissue utilized in “Methodology for intra-species analysis” section for human as well as rhesus. In this case, human samples (including both 5mC and 5hmC samples) are considered as control samples, whereas rhesus samples (including both 5mC and 5hmC samples) are used as experimental samples.

### Notification of differentially multi-stage methylated genes

Since it is already mentioned in “Methodology for intra-species analysis” section that no statistical hypothesis test is appropriate for very small sample sizes, therefore fold-change technique is applied in this inter-species analysis on the common genes for human and rhesus in order to determine differentially multi-stage methylated genes since here number of samples is very small.

Fold change for each gene for inter-species study (viz., *FC*_*inter*_) is defined as the fraction of the mean of all the samples of rhesus (including both the 5hmC and 5hmC samples) to the mean of all the samples of human (including both the 5mC and 5hmC samples).

6 $$ {FC}_{inter}(i)= \frac{\bar{y}_{5mC\_5hmC}^{rhesus}(i)}{\bar{y}_{5hmC\_5hmC}^{human}(i)},  $$

where $\bar {y}_{5mC\_5hmC}^{rhesus}(i)$ stands for the mean of the values across all the samples of rhesus (including both the 5mC and 5hmC samples), and $\bar {y}_{5mC\_5hmC}^{human}(i)$ stands for the mean of the values across all the samples of human (including both the 5mC and 5hmC samples).

For this inter-species study between human and rhesus, we also set a same threshold. The resulting list of the differentially multi-stage methylated genes for this inter-species study is symbolized as *DM*2^*humanrhesus*^.

### Computing multi-stage co-methylation using weighted TOM

For determining the correlation in terms of multi-stage methylation levels for inter-species study, we again start with Pearson’s correlation between pairwise genes belonging to *DM*2^*humanrhesus*^. Thereafter, we utilize weighted topological matrix as a weighted connectivity measure for the geneset *DM*2^*humanrhesus*^ where Pearson’s correlation scores are treated as the weighted-values for adjacency matrix.

The mentioned weighted topological matrix score between two vertices *i* and *j* for inter-species study (viz., *TOM*_*inter*_(*i*,*j*)) can be stated as follows.

7 $$ {TOM}_{inter}(i,j) =\left\{\begin{array}{lc} \frac{\sum\limits_{v\neq i,j} Ad(i,v) Ad(j,v) + Ad(i,j)}{min\left\{\sum\limits_{v\neq i}Ad(i,v), \sum\limits_{v\neq j}Ad(j,v)\right\}- Ad(i,j)+1}, & \text{if \(i\neq j\)},\\ 1, & \text{if i==j}, \end{array}\right.  $$

where *Ad* denotes weighted adjacency matrix of the nodes having Pearson’s correlation values as the weights for the inter-species study.

We thereafter compute the corresponding dissimilarity scores of *TOM*_*inter*_ values (similarity scores).

8 $$ {dissim}_{inter}(i,j) = 1 - {TOM}_{inter}(i,j).  $$

Average linkage clustering [29] is then brought into service on genes using *dissim*_*inter*_(*i*,*j*) scores that is demonstrated as follows.

9 $$ L_{inter}(s,t)=\frac{1}{n_{s} n_{t}} \sum\limits_{i \in C_{s}} \sum\limits_{j \in C_{t}} De(y_{i}, y_{j}),  $$

where

10 $$ De(i,j)={dissim}_{inter}(i,j).  $$

Here, *s* and *t* are two clusters made for inter-species, De(.,.) stands for distance between a point of first cluster and a point of second cluster for inter-species, and *L*_*inter*_(.,.) denotes the distance between two clusters using average linkage clustering for inter-species. Then we get corresponding dendrogram of the clusters. Dynamic tree cut [30] is then utilized on the dendrogram using color-thresholding method. As a result, inter-species multi-stage co-methylated gene-modules are determined. These inter-species multi-stage co-methylated gene-modules are termed as *GM*2^*humanrhesus*^.

### TF-miRNA-gene networks analysis for inters-species

We individually perform KEGG pathway and gene-ontology analyses using DAVID database on the genes of each module belonging to *GM*2^*humanrhesus*^ for the purpose of verifying them with existing pathway evidences. Additionally, we determine TFs using ITFP and Transfac databases [27] that regulate the genes belonging to *DM*2^*humanrhesus*^. We also determine miRs using miRTarBase and PITA databases [27] which target the genes of *DM*2^*humanrhesus*^.

**Fig. 2:**
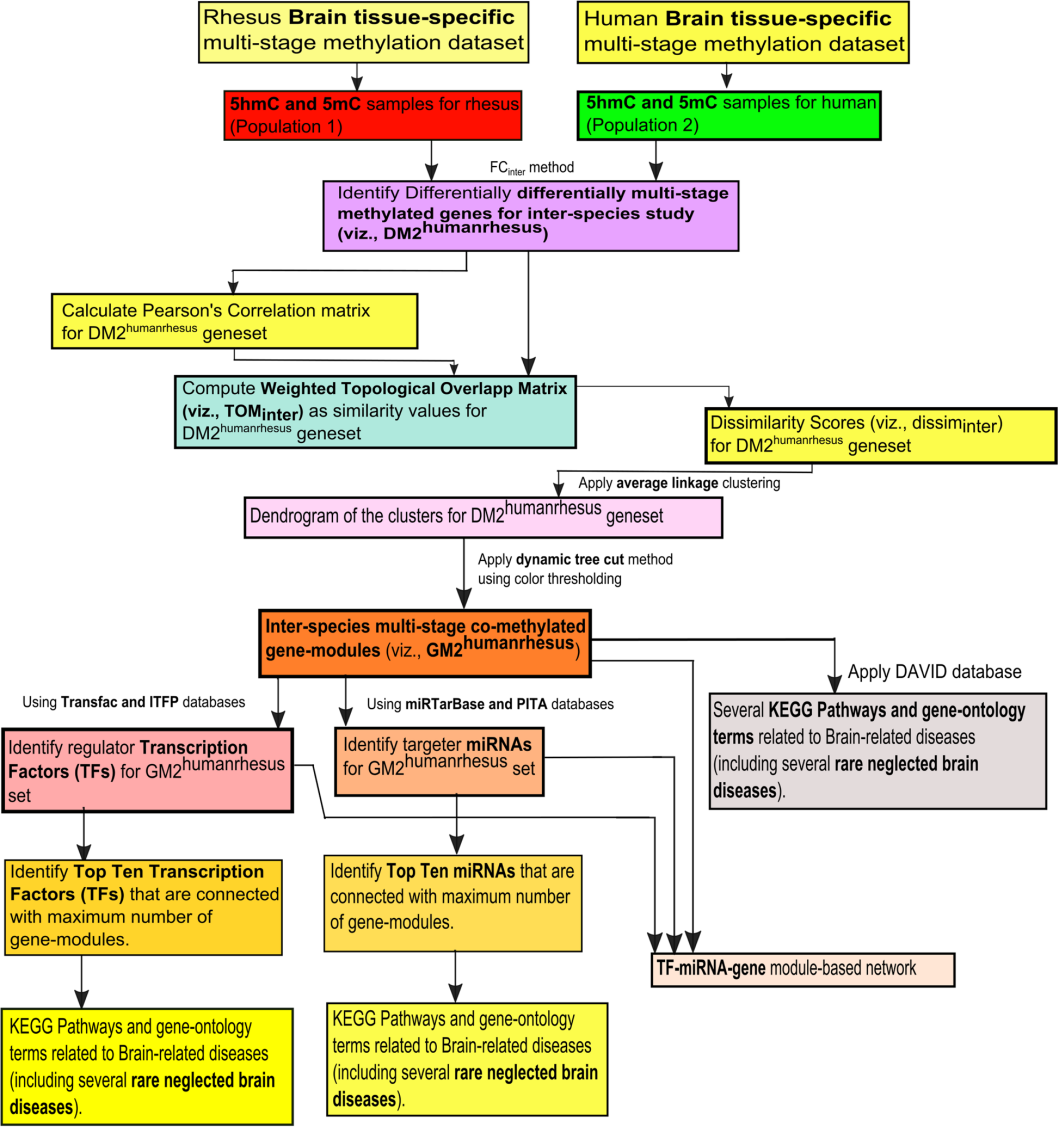
Flowchart of the proposed framework of gene-module detection for inter-species brain-specific multi-stage methylated dataset between human and rhesus

The top ten regulator TFs that associate with maximum gene-modules, are identified; whereas top ten targeter miRNAs that link with maximum gene-modules, are determined. It is noted that when more than one TF/miRNA are connected to same number of gene-modules, then these TFs/miRNAs are further ranked in descending order of the maximum number of participating regulated/targeted genes from the different modules. However, these top ten TFs/miRNAs are then verified by literature search for recognizing how many of them have an association with the corresponding diseases related to the sample tissue. The framework of gene-module detection for the inter-species study between the rhesus and human is represented in Fig. 2.

### Dataset

For this study, we utilize a methylation dataset (NCBI ref. id.: GSE49177) [25] having both 5hmC and 5hmC samples for brain tissue of human as well as rhesus. For human, there are six 5hmC samples and three 5hmC samples for 4,85,577 gene probes; whereas for rhesus, eleven 5hmC samples and seven 5hmC samples exist for 4,85,577 gene probes.

## Results and discussion

In this section, initially we brief about the experimental dataset, and thereafter list up the outcomes after following the proposed frameworks highlighted in Figs. 1 and 2. The sets of results are different depending on intra and inter species analyses. By following the dataset and methodological frames, the results are distributed in several subsections.

### Results for intra-species analysis

As mentioned in “Notification of differentially 5hmC-methylated genes” section, we identify a total of 598 common differentially 5hmC-methylated genes for both rhesus and human (i.e., $DM^{human}_{5hmC}$ for human, and $DM^{rhesus}_{5hmC}$ for rhesus). Thereafter, weighted *TOM* is computed for each gene-pair belonging to the gene-list $DM^{human}_{5hmC}$ as well as the gene-list $DM^{rhesus}_{5hmC}$ individually, where Pearson’s correlation scores are used in weighted adjacency matrix. The dissimilarity values are then calculated from the weighted *TOM* values. Using these dissimilarity values, we perform average linkage clustering and extract the corresponding dendrogram. Thereafter, dynamic tree cut algorithm is applied to identify the color specific highly correlated modules with highly correlated genes.

#### For human

For human, color-specific modules with corresponding dendrogram structures of the highly similar intra-species co-methylated gene-modules (i.e., $GM^{human}_{5hmC}$) is constructed. In case of $GM^{human}_{5hmC}$, we identify five different color modules which are represented in blue, brown, green, turquoise and yellow, individually (depicted in Table 1). The number of genes in each of these five modules are 102, 98, 3, 121 and 8, respectively.Using KEGG pathway and gene-ontology analyses through DAVID database, the genes from different modules are verified. After having these color modules, we determine the corresponding regulator TFs and targeter miRNAs of the above resulting genes belonging to different modules for human as well as rhesus individually using the databases mentioned in [27]. For human, 497 miRs and 127 TFs are identified. Thereafter, a corresponding TF-miRNA-gene network for human intra-species study has been created. Among them, the list of top ten TFs are then selected from the complete TF-list (Additional file 1) depending upon the maximum number of associated gene-modules. Similarly, the list of top ten miRs are then chosen from the complete list (Additional file 1) depending on the maximum number of connected gene-modules. Notably, the TF/miRNA, connected with same number of gene-modules have further been ranked in decreasing order of the maximum number of participating regulated/targeted genes from the different modules. For human, there are two tables (viz., Tables 3 and 4) that provide the lists of top ten TFs and top ten miRs, respectively. Among the top ten TFs for human represented in Table 3, the number of known/verified TFs is eight, whereas remaining two TFs are novel (viz., **MYST3** and **ZNF771**). Similarly, among the top ten miRNAs, all are known for human brain tissue (presented in Table 4).

**Table 1:** List of five detected color-modules of genes for the intra-species analysis of human samples by the proposed method with related biologically and statistically information and related disease identification

Sl. No	Module color	Gene	Related major significant KEGG pathways (*p*-value) (diseases)	Related major signifiacnt GO-terms (*p*-value) (diseases)	miR (targeter)	TF (targeter)
1	Blue	102	-	-	276	56
2	Brown	98	Cell adhesion molecules (4.0E-2) [72] (Neuro disease [61]).	GO:CC:- GO:0005886 plasma membrane (1.0E-2) GO:MFs:- GO:0003774 motor activity (3.0E-2), (motor neuron degeneration [62]), GO:0003700 transcription factor activity (4.1E-2) (Hungtington’s Disease [63])	78	36
3	Green	3	-	-	31	2
4	Turquoise	121	-	GO:BP:- GO:0051252 regulation of RNA metabolic process (1.7E-3). GO:MFs:- GO:0003677 DNA binding (2.4E-3), GO:0003700 transcription factor activity (2.7E-2).	112	40
5	Yellow	8	mTOR signaling pathway (0.032929).	-		

#### For Rhesus

The color-specific modules with corresponding dendrogram structures of the highly similar intra-species co-methylated gene-modules for rhesus (viz., $GM^{rhesus}_{5hmC}$) is constructed. Two modules, namely blue and turquoise, are same as that of human samples, have been found for $GM^{rhesus}_{5hmC}$ (represented in Table 2). The number of genes in each of these two modules are 73 and 349, respectively. Using KEGG pathway and gene-ontology analyses through DAVID database, the genes from different modules are verified (a case study where 5hmC is controlled and 5mC is diseased are mentioned in Additional files 2 and 3). In Table 5, the list of top ten such TFs for rhesus has been provided, whereas in Table 6, the list of top ten such miRs is represented for rhesus (complete list is given at Additional file 4). Infact, we performed literature search on these top ten TFs/miRNAs to identify how many of them are associated with the corresponding diseases related to the sample tissue (brain tissue here). While in the search search for Intra-Species analysis, we have found some of the important observations for the intra-species analysis as well. We have detected some of the **rare diseases** like **HiV mediated dementia**, **SIV mediated enchaphilits** etc for human as well as rhesus. For rhesus, seven TFs are verified or previously discovered with relevant disease area, whereas remaining three TFs (viz., **BAZ2B**, **RCOR3** and **ATF1**) among them remain novel (depicted in Table 5). **mml-hsa-np** and **mml-miR-561** are remained novel miRNAs among the top ten miRNAs (presented in Table 6).

**Table 2:** List of two detected color-modules of genes for the intra-species analysis of rhesus samples by the proposed method with related biologically and statistically information

Sl. No	Module color	Gene	Major significant KEGG pathways (*p*-value) (diseases)	miR (targeter)	TF (targeter)
1	Blue	73	-	193	41
2	Turquoise	349	Neuroactive ligand-receptor interaction (3.6E-2) [72] (Cortical synaptic development related disease [60]	374	118

### Discussion on intra-species analysis

We recognize some top TFs/miRNAs/gene-modules that are responsible for some known diseases as well as several types of rare **neurodegenerative diseases** (NDs). In case of human, for example, according to [31], the topmost TF WHSC1 is liable for the disease wolf Hirschhorn syndrome; whereas the next TF E2F4 is connected with Neuro-degenerative disorder [32]. Interestingly, according to [33], TF EPAS1 is associated with disorder, i.e., “Iron toxicity effected the aging of brain”. Besides these, the TFs namely ZBTB16, EZF1, TRMT1 and SSRP1 share connection with the diseases namely Hungtinton’s Disease [34], Parkinson’s Disease [35], Oxidative damage in Neurodegenrative diseases [36] and Alzheimer’s Disease [37], respectively. Table 3 demonstrates these detailed information regarding the involvement of the top TFs with diseases for human intra-species study. On the other hand, as mentioned in [38], the topmost miRNA namely hsa-miR-579 has a connection with Alzheimer’s Disease; whereas the second top miRNA entitled as hsa-miR-495 relates to HIV mediated dementia [39]. Furthermore, hsa-miR-34a, hsa-miR-34c-5p, hsa-miR-449a, hsa-miR-607 and hsa-miR-664-3p are associated with neuropsychaitric and nuerodegenrative diseases [40], Bipolar disorder and Schizophenia [41], Molecular Targets for Neurodegenerative diseases [42], Neurodegenerative diseases [43], and Psychiatric and Neurodegenerative disease [44], respectively. Table 4 describes the whole information regarding the relation between the top miRNAs and diseases for human intra-species study.

**Fig. 3:**
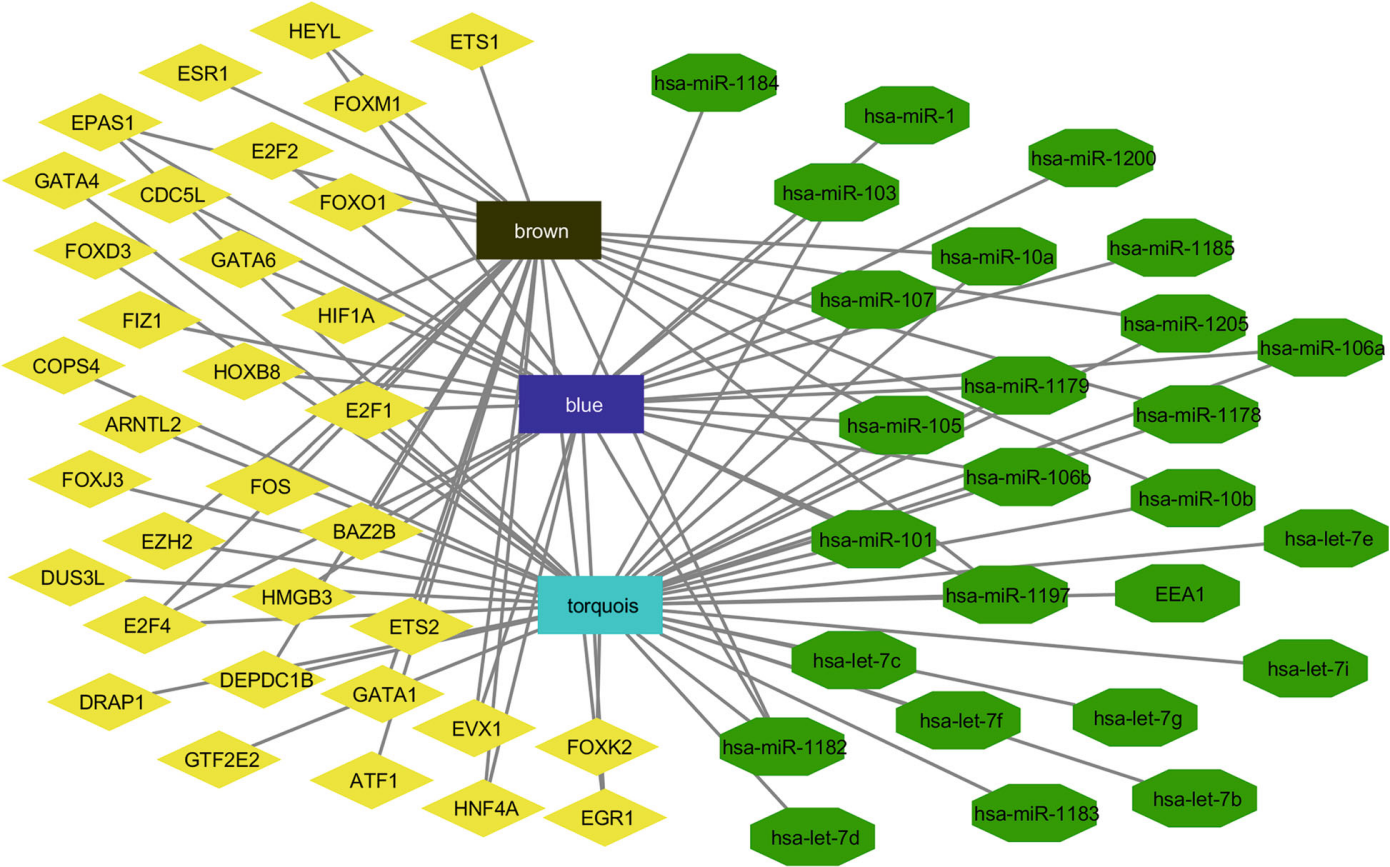
Tf-color modules-MicroRNAs network for rhesus monkey where diamond represents TFs, pentagon represents MicroRNAs and square represents gene specific color modules

**Fig. 4:**
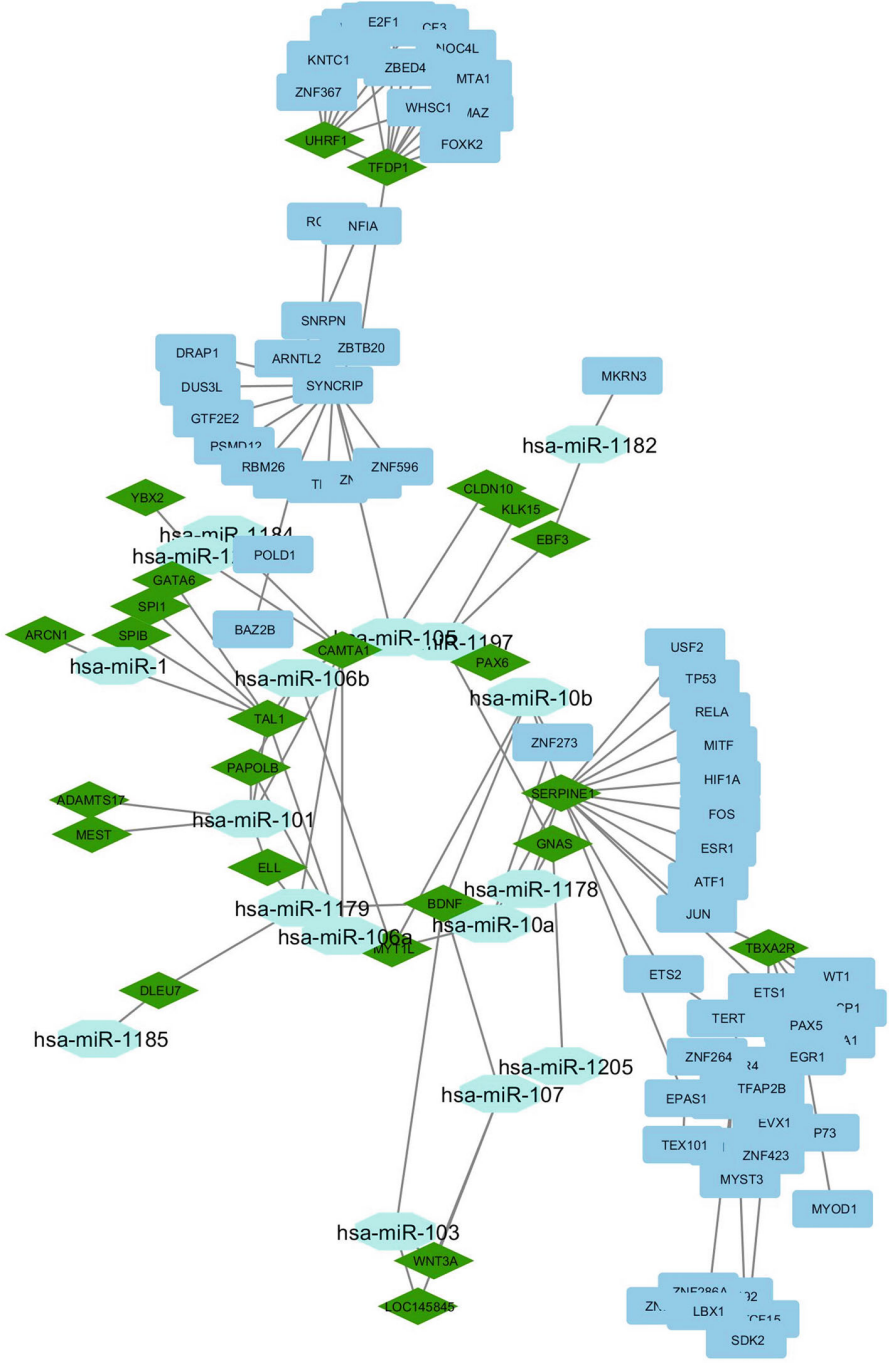
Tf-Gene-MicroRNAs network for rhesus monkey where diamond represents TFs, pentagon represents MiRs and square represents gene modules

In case of rhesus, for example, the TFs namely E2F4, ZBTB20, FOXM1, SSRP1, TEDP1, ZNF207 and MYBL2 are connected with the diseases namely SIV encephalitis [45], Age associated brain disease [46], Glioma [47], Kaposi’s Sarcoma [48], Accute or chronic neurodegenerative diseases [49], Epigenetic regulation of puberty [50], and Kaposi Sarcoma and effecting sexual growth [51], respectively. We have shown the top TFs and its related diseases for rhesus inter-species in Table 5. Besides these, mml-miR-338-5p, mml-miR-218, mml-miR-495, mml-miR-203, mml-miR-590-3p, mml-miR-596, mml-miR-548p, and mml-miR-524-5p have connection with “an auto immune disease like SIV” [52], Huntintong’s Disease [53], Age mediated skeletal muscle contamination [54], Enhances Coxsackievirus B3 replication [55], Neuro inflammation Disorder [56], Schizophernia [57], monkey hippocampus effected [58], and Amyotrophic lateral sclerosis [59], respectively. Table 6 describes the details information about the relation between the top miRNAs and diseases for rhesus intra-species study. In Fig 3, a network is created with color modules which are targeted by miRs and regulated by TFs. Similarly, a network is created with genes from different modules which are targeted by miRs and regulated by TFs for rhesus (Fig. 4).

**Table 3:** Top ten targeter TFs for Human (that are connected with maximum number of gene-modules and that regulate maximum number of genes from the connected gene-modules) with information of their maximum number of regulated genes as well as gene-modules, and verified information of them

SL. No	TFs	Number of connected gene-modules	Number of regulated genes	Name of the connected gene-modules (number of regulated genes in the modules)	Disease and Disease-verified information of TFs	Status of TFs
1	WHSC1	3	9	Blue (4), Brown (2), Turquoise (3).	wolf Hirschhorn syndrome [31]	Known
2	E2F4	3	7	Blue (4), Brown (2), Turquoise (1).	Neuro degeneration disorder [32]	Known
3	EPAS1	3	5	Blue (2), Brown (2), Turquoise (1).	Iron toxicity effected the aging of brain [33]	Known
4	LBX1	3	5	Blue (2), Brown (2), Turquoise (1).	Septo optic Dysplasia [73]	Known
5	MYST3	3	5	Blue (2), Brown (2), Turquoise (1).		Unknown
6	ZNF771	3	4	Blue (2), Brown (1), Turquoise (1).	-	Unknown
7	ZBTB16	3	3	Blue(1), Brown (1), Turquoise (1).	Hungtinton’s Disease [34]	Known
8	EZF1	3	3	Blue (1), Brown (1), Turquoise (1)	Parkinson’s Disease [35]	known
9	TRMT1	3	3	Blue (1), Brown (1), Turquoise (1).	Oxidative damage in Neurodegenrative diseases [36]	Known
10	SSRP1	3	3	Blue (1), Brown (1), Turquoise (1).	Alzheimer’s Disease [37]	Known

**Table 4:** Top ten targeter miRNAs for Human (that are connected with maximum number of gene-modules and that target maximum number genes from the connected modules) with information of their maximum number of targeted genes as well as gene-modules, and verified information of them

Sl. No.	MiRNAs	Number of connected gene-modules	Number of targeted genes	Name of the connected gene-modules (number of targeted genes in the modules)	Disease and Disease-verified information of miRNAs	Status of miRNAs
1	hsa-miR-579	4	17	Blue (2), Brown (2), Green (1), Turquoise (12).	Alzheimer’s Disease [38]	Known
2	hsa-miR-495	4	14	Blue (5), Brown (2), Green (1), Turquoise (6).	HIV mediated dementia [39]	Known
3	hsa-miR-27b-3p-3p	4	10	Blue (5), Brown (1), Green (1), Turquoise (3).	Presence in high intensity for Alzheimer’s disease [74]	Known
4	hsa-miR-449b-5p-5p	4	10	Blue (1), Brown (2), Green (1), Turquoise (6).	Presence in low intensity in case of Alzheimer’s disease	Known
5	hsa-miR-34a	4	10	Blue (1), Brown (2), Green (1), Turquoise (6).	neuropsychaitric and nuerodegenrative diseases [40]	Known
6	hsa-miR-34c-5p	4	10	Blue (1), Brown (2), Green (1), Turquoise (6).	Bipolar disorder and Schizophenia [41]	Known
7	hsa-miR-449a	4	10	Blue (1), Brown (2), Green (1), Turquoise (6).	Molecular Targets for Neurodegenerative Diseases [42]	Known
8	hsa-miR-607	4	9	Blue (3), Brown (3), Green (1), Turquoise (2).	Neurodegenerative diseases [43]	Known
9	hsa-miR-27a-3p	4	9	Blue (4), Brown (1), Green (1), Turquoise (3).	Alzheimer’s Disease [75]	Known
10	hsa-miR-664-3p	4	9	Blue (2), Brown (2), Green (1), Turquoise (4).	Psychiatric and Neurodegenerative disease [44].	Known

**Table 5:** Top ten targeter TFs for Rhesus monkey (that are connected with maximum number of gene-modules and that regulate maximum number of genes from the connected gene-modules) with information of their maximum number of regulated genes as well as gene-modules, and verified information of them

SL. No.	TFs	Number of connected gene-modules	Number of regulated genes	Name of the connected gene-modules (number of regulated genes in the modules)	Disease and Disease-verified information of TFs	Status of TFs
1	BAZ2B	2	11	Blue (9), Turquoise (2).		Unknown
2	E2F4	2	10	Blue (2), Turquoise (8).	SIV encephalitis [45]	Known
3	ZBTB20	2	10	Blue (5), Turquoise (5).	Age associated brain disease [46]	Known
4	FOXM1	2	8	Blue (2), Turquoise (6).	Glioma [47]	Known
5	RCOR3	2	7	Blue (6), Turquoise (1).	-	Unknown
6	SSRP1	2	7	Blue (4), Turquoise (3).	Kaposi’s Sarcoma [48]	Known
7	TEDP1	2	6	Blue (3), Turquoise (3).	Accute or chronic neurodegenerative diseases [49]	Known
8	ATF1	2	6	Blue (5), Turquoise (1).	-	Unknown
9	ZNF207	2	5	Blue (2), Turquoise (3).	Epigenetic regulation of puberty [50]	Known
10	MYBL2	2	5	Blue (2), Turquoise (3).	Kaposi Sarcoma and effecting sexual growth [51]	Known

**Table 6:** Top ten targeter miRNAs for Rhesus monkey (that are connected with maximum number of gene-modules and that target maximum number genes from the connected modules) with information of their maximum number of targeted genes as well as gene-modules, and verified information of them

Sl. No.	MiRNAs	Number o connected gene-modules	Number of targeted genes	Name of the connected gene-modules (number of targeted genes in the modules)	Disease and Disease-verified information of miRNAs	Status of miRNAs
1	mml-miR-338-5p	2	33	Blue (6), Turquoise (27).	An auto immune disease like SIV [52]	Known
2	mml-miR-218	2	25	Blue (6), Turquoise (19).	Huntintong’s Disease [53]	Known
3	mml-miR-768-3p	2	25	Blue (9), Turquoise (16).		Unknown
4	mml-miR-495	2	24	Blue (10), Turquoise (14).	Age mediated skeletal muscle contamination [54]	Known
5	mml-miR-203	2	24	Blue (6), Turquoise (18).	Enhances Coxsackievirus B3 replication [55]	Known
6	mml-miR-561	2	24	Blue (11), Turquoise (13).		Unknown
7	mml-miR-590-3p	2	23	Blue (6), Turquoise (17).	Neuro inflammation Disorder [56]	Known
8	mml-miR-596	2	23	Blue (2), Turquoise (21).	Schizophernia [57]	Known
9	mml-miR-548p	2	22	Blue (11), Turquoise (11).	monkey hippocampus effected [58].	Known
10	mml-miR-524-5p	2	22	Blue (8), Turquoise (14).	Amyotrophic lateral sclerosis [59].	Known

According to Table 3, the TF WHSC1 regulates a total of nine genes from different gene-modules out of which four genes (viz., KIFC1b, KIFC1a, MSI2 and KIFC1) belong to blue module, two genes (i.e., UHRF1 and MYH11) are the part of brown module, and remaining three genes (namely, GTF2H4, TFDP1 and UHRF1a) are in turquoise module. As mentioned in Table 4, the miRNA hsa-miR-579 targets a total of seventeen genes from different gene-modules out of which two genes (i.e., SPRY2 and YBX2) belong to blue module, another two genes (i.e., two specific GNAS family proteins) are coming from brown module, one gene (viz., C1orf144) is from green module, and remaining twelve genes (namely, rest of the GNAS family proteins) exist in turquoise module.

**Fig. 5:**
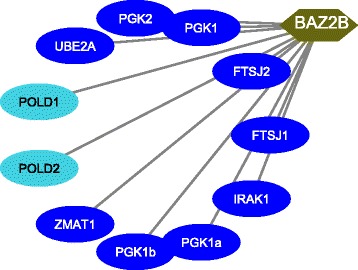
Top TF for rhesus (in Intra-species study mentioned in Table 3) and its targeted genes from different modules

**Fig. 6:**
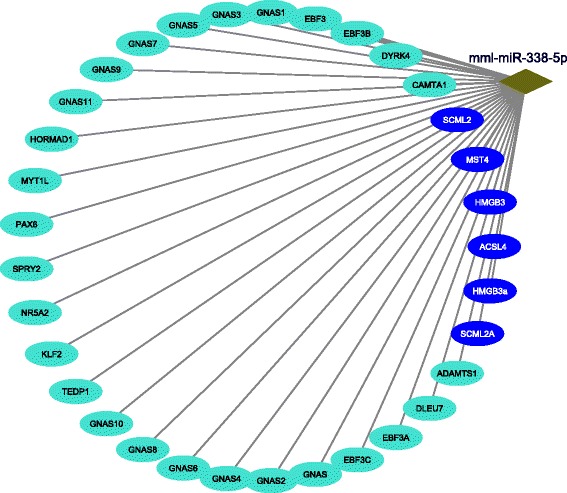
Top MiRNA for rhesus (in Intra-species study mentioned in Table 6) and its targeted genes from different modules

Table 2 shows the detected intra-species co-methylated modules for rhesus (i.e., Blue and Turquoise). Blue module consists of 73 genes, whereas Turquoise modules has 349 genes. A total of one hundred ninety three miRNAs target the different genes of the Blue module, whereas forty one TFs regulate different genes of the Blue module. For Turquoise module, a total of three hundred seventy four miRNAs target different genes belonging to that module, whereas one hundred eighteen TFs regulate the different genes of that module. In case of the Turquoise module, we identify an important KEGG pathway, viz., Neuroactive ligand-receptor interaction (*p*-value= 3.6E-2) in which several genes of the module are significantly involved. Notably, Liu et al. [60] supports that above pathway is associated with cortical synaptic development related diseases. A subnetwork that consists of the topmost TF (i.e., BAZ2B) and its regulated genes for rhesus intra-species analysis, is presented by Fig. 5. According to Table 5 and Fig. 5, BAZ2B regulates eleven genes from different modules of which nine genes (i.e., UBE2A, PGK2, PGK1, FTSJ family proteins, IRAK1, PGK1b, PGK1a, ZMAT1) belong to blue module whereas remaining two genes (viz., POLD1 and POLD2) exist in turquoise module. Fourth subnetwork is depicted by Fig. 6 in which there are the topmost miRNA (namely, mml-miR-338-5p) and its targeted genes for rhesus intra-species analysis. As highlighted in Table 6 and Fig. 6, among the total thirty three genes from different modules targeted by mml-miR-338-5p, twenty seven genes (i.e., ADAMTS1, DLEU7, EBF3 family proteins, HORMAD1, MYT1L, QAX6, SPRY2, NR5A2, KLF2, GNAS family proteins, DYRK4, CAMTA1) come from turquoise module and remaining six genes (viz., MST4, ACSL4, HMGB3 family proteins, SCML2 family proteins) belong to blue module.

Table 1 shows the detected intra-species co-methylated modules for human (i.e., Blue, Brown, Green, Turquoise and Yellow). The number of existing genes in the Blue, Brown, Green, Turquoise and Yellow modules are 102, 98, 3, 121 and 8, respectively. For Blue module, 276 miRNAs target different genes of the module, and 56 TFs regulate different genes of the Blue module. For Brown module, a total of 78 miRNAs target different genes of the module, whereas 36 TFs regulate different genes of the Brown module. Here, we identify an important KEGG pathway viz., cell adhesion molecules (*p*-value = 4.0E-2). Notably, Wei et al. [61] supports that this KEGG pathway is associated with neuro diseases. Along with, several other gene-ontology terms are related to the genes of the Brown module, viz., GO:CC of plasma membrane (*p*-value = 1.0E-2), and GO:MFs of motor activity (*p*-value = 3.0E-2) and transcription factor activity (*p*-value = 4.1E-2). Here, GO:MF of motor activity is involved with motor neuron degeneration [62], whereas GO:MF of transcription factor activity is related to Hungtington’s disease [63]. For Green module, 31 miRNAs target the different genes of the module, whereas 2 TFs regulate the different genes of the green module. Similarly, 112 miRNAs target different genes of the Turquoise module, whereas 40 TFs regulate different genes of the Turquoise module. Several gene ontology terms are associated with genes of the Turquoise module. Those are GO:BP of regulation of RNA metabolic process (*p*-value = 1.7E-3), GO:MFs of DNA binding (*p*-value = 2.4E-3), and Transcription factor activity (*p*-value = 2.7E-2). In case of yellow module, we identify an important pathway viz., mTOR signaling pathway (*p*-value = 0.032929).

### Results and discussion for inter-species analysis

**Fig. 7:**
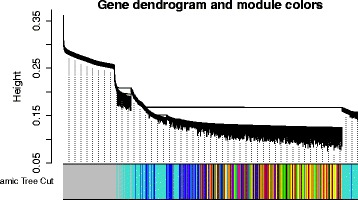
Dendrogram and corresponding color modules for rhesus over human from inter-species analysis

As mentioned in “Notification of differentially multi-stage methylated genes” section, we determine 598 common genes as *DM*2^*humanrhesus*^ genes for both human and rhesus species. Thereafter, Pearson’s correlation score is calculated for each gene-pair belonging to *DM*2^*humanrhesus*^. Weighted *TOM* is then performed as a weighted connectivity measure where the aforementioned Pearson’s correlation scores are utilized as weighted values of the adjacency matrix. The corresponding dissimilarity values are then computed from the Weighted *TOM* (similarity) values. Using those dissimilarity values, a dendrogram is constructed by average linkage clustering. Dynamic tree cutter method is applied with color thresholding. As a result, we identify eighteen inter-species multi-stage co-methylated modules (denoted as *GM*2^*humanrhesus*^). See Fig. 7 for this. These modules are colored by black, blue, brown, cyan, green, greenyellow, grey60, lightcyan, lightyellow, magenta, midnightblue, pink, purple, red, salmon, tan, turquoise and yellow. In Table 7, details about the modules of inter-species analysis have been provided.

**Table 7:** List of eighteen detected color-modules of genes for the inter-species analysis of human and rhesus samples by the proposed method with related biologically and statistically information

Sl. No	Module color	Gene	Related major significant KEGG pathways (*p*-value)	Related major signifiacnt GO-terms (*p*-value)	miR (targeter)	TF (targeter)
1	Black	65	Alzeimer’s disease (8.41E-13), Neuro-trophin Signaling pathway (5.46E-6)	GO:CC:- GO:0014069 Postsynaptic Density (2.3E-2).	66	25
				GO:MFs:- GO:0060589 zinc ion binding (1.7E-3), cytokine binding (0.00895).		
2	Blue	218	Tight junction (1.1E-2), Phosphatidylinositol signaling system (2.0E-2)	GO:BPs:- GO:0035556 Intracellular signaling transduction (4.2E-4), GO:0000902 cell morphogenesis (2.7E-3)	83	71
				GO:CCs:- GO:0014069 Synaptosome (4.7E-3), GO:0035097 histone methyl transferase complex (2.9E-2).		
3	Brown	167	-	GO:BPs:-GO:0048666 Neuron development (2.2E-2), GO:0000902 cell morphogenesis (2.6E-2).	53	59
				GO:CC:- intracellular organelle lumen (0.019554).		
				GO:MFs:- protein kinase activity (1.82E-08), protein serine/threonine kinase activity (4.33E-07), MAP kinase tyrosine/serine/threonine phosphatase activity (0.004947), protein tyrosine kinase activity (0.012018).		
4	Cyan	1	-	-	5	4
5	Green	63	-	GO:CCs:- GO:0044459 plasma membrane part (2.8E-2), GO:0044431 golgi apparatus part (4.4E-2).	27	9
6	Greenyellow	4	-	-	6	4
7	Grey60	2	-	-	17	3
8	Lightcyan	2	-	GO:BPs:- GO:0048754 branching morphogenesis of an epithelial tube (4.8E-3), GO:0001525 angiogenesis (1.1E-2)..	14	1
9	Lightyellow	3	B-cell receptor signaling pathway (2.9E-2), T cell receptor signaling pathway (4.2E-2).	GO:BPs:- negative regulation of transport (2.0E-2), regulation of cellular localization (3.6E-2).	26	1
10	Magenta	16	-	GO:CC:- GO:0045202 synapse (3.6E-2).	3	6
11	Midnightblue	2	-	-	1	1
12	Pink	4	-	GO:BP:- GO:0014070 response to organic cyclic substance (1.80E-2).	3	0
13	Purple	9	-	-	0	5
14	Red	89	-	-	80	11
15	Salmon	1	-	-	44	2
16	Tan	2	-	-	-	-
17	Turquoise	451	adherens junction (3.0E-2)	GO:BPs:- negative regulation of signal transduction (7.7E-3), negative regulation of cell communication (1.1E-2). GO:CCs:- neuron projection (3.4E-2). GO:MFs:- protein complex binding (6.3E-3), SMAD binding (1.3E-2), DNA binding (4.5E-2), enzyme binding (4.6E-2), ion binding (4.9E-2).	250	130
18	Yellow	2	MAPK signaling pathway (1.5E-2).	GO:BPs:- hindbrain morphogenesis (2.3E-2), hindbrain development (2.4E-2), Ras protein signal transduction (2.3E-2). GO:CCs:- cell junction (4.9E-5), synapse (2.4E-2), postsynaptic density (3.3E-2). GO:MFs:- GTPase regulator activity (2.1E-5), transcription factor binding (4.3E-2).	62	82

After obtaining these color modules, we use different databases to determine TFs and miRNAs (miRs) that regulate and target genes from different color modules respectively. We utilize Transfac and ITFP databases [27] to identify the regulator TFs for corresponding genes, and also use miRTarBase and PITA database to determine targeter miRs for corresponding genes. For verification purpose, we perform a comprehensive literature search, and subsequently, we carry out KEGG pathway and gene-ontology analyses using DAVID database. Among all these modules, Turquoise module is most dense module consisting of 451 genes. In Table 8, the list of top ten TFs for both human and rhesus monkey that are connected with maximum number of gene-modules among the resultant modules, is identified for inter-species study, whereas in Table 9, the list of top ten miRs for both human and rhesus monkey that are connected with maximum number of gene-modules among the resultant modules, is determined for inter-species analysis. Notably, when more than one TF/miRNA are connected with same number of gene-modules, then these TFs/miRNAs have further been ranked in decreasing order of the maximum number of participating regulated/targeted genes from the different modules.

**Table 8:** Top ten targeter TFs for both Human and Rhesus monkey (that are connected with maximum number of gene-modules and that regulate maximum number of genes from the connected gene-modules) with information of their maximum number of regulated genes as well as gene-modules, and verified information of them for inter-species analysis

SL. No.	TFs	Number of connected gene-modules	Number of regulated genes	Name of the connected gene-modules (number of regulated genes in the modules)	Disease-verified information of TFs for human	Status of TFs for human	Disease-verified information of TFs for rhesus	Status of TFs for rhesus
1	ZNF286A	10	31	Black(1), Blue (2), Brown (3), Cyan (1), Green (6), Magenta (1), Purple (1), Red (2), Turquoise (7), Yellow (7).	Identification and analysis of plasticity induced late response [64]	known	Hemi-parkinson’s disease [65]	Known
2	E2F4	9	61	Black(3), Blue (14), Brown (13), Green (4), Magenta (1), Midnightblue(1), Pink(2) Turquoise (15), Yellow (8).	Neurodegeneration disorder [32]	Known	SIV encephalitis [45]	Known
3	ZNF771	9	46	Black(2), Blue (2), Brown (7), Cyan (1), Green (5), Lightcyan(1), Red (4), Turquoise (16), Yellow (8).	-	Unknown	-	Unknown
4	ZNF423	9	42	Black(2), Blue (2), Brown (3), Cyan (1), Green (6), Purple (1), Red (2), Turquoise (15), Yellow (7).	Joubart’s Syndrome [67]	Known	-	Unknown
5	SP1	9	41	Black(1), Blue (7), Brown (5), Green (3), Lightcyan(1), Purple (1), Red (2), Turquoise (15), Yellow (7).	Disrupt in early Huntington’s disease [63]	Known	mediate members of brain aging proteins and enhancing the chances of Alzeimer’s Disease [66]	Known
6	ZBTB20	7	42	Black(2), Blue (12), Brown (3), Green (1), Red (1), Turquoise (17), Yellow (6).	Major depressive disorder [68]	Known	Age associated brain disease [46]	Known
7	SP3	7	29	Black(2), Blue (3), Brown (3), Green (4), Red (3), Turquoise (10), Yellow (4).	Neuro degeneration disease [69]	Known	-	Unknown
8	LBX1	7	28	Black(1), Brown (5), Cyan (1), Green (5), Red (3), Turquoise (7), Yellow (6).	Septo optic dysplasia [73]	Known	-	Unknown
9	MYST3	7	24	Black(1), Blue (3), Brown (3), Green (1), Red (1), Turquoise (10), Yellow (5).	-	Unknown	-	Unknown
10	FOXM1	7	19	Black(1), Blue (5), Brown (5), Green (1), Red (1), Turquoise (4), Yellow (2).	Glioma [47]	Known	-	Unknown

**Table 9:** Top ten targeter miRNAs for both Human and Rhesus monkey (that are connected with maximum number of gene-modules and that target maximum number genes from the connected modules) with information of their maximum number of targeted genes as well as gene-modules, and verified information of them for inter-species analysis

Sl. No.	MiRNAs	Number of connected gene-modules	Number of targeted genes	Name of the connected gene-modules (number of targeted genes in the modules)	Disease-verified information of TFs for human	Status of TFs for human	Disease-verified information of TFs for rhesus	Status of TFs for rhesus
1	miR-19b	14	121	Black (8), Blue (18), Brown (9), Green (3), Gray60 (1), Lightyellow (1), Magenta (2), Pink (1), Purple (1), Red (9), Salmon (1), Tan (1), Turquoise (45), Yellow (21).	associated with Alzeimer’s disease [71]	Known	-	Unknown
2	miR-19a	14	119	Black (8), Blue (18), Brown (8), Green (3), Gray60 (1), Lightyellow (1), Magenta (2), Pink (1), Purple (1), Red (9), Salmon (1), Tan (1), Turquoise (44), Yellow (21).	HIV mediated neurodegenerative disease [39]	Known	-	Unknown
3	miR-520d-5p	12	130	Black (8), Blue (14), Brown (21), Green (4), Greenyellow(1), Magenta (2), Pink (1), Purple (1), Red (8), Tan (1), Turquoise (52), Yellow (17).	Alzeimer’s disease [76]	Known	-	Unknown
4	miR-524-5p	12	130	Black (8), Blue (14), Brown (21), Green (4), Greenyellow(1), Magenta (2), Pink (1), Purple (1), Red (8), Tan (1), Turquoise (52), Yellow (17).	neuroblastoma [77]	Known	Amyotrophic lateral sclerosis [59].	Known
5	miR-519b-5p	12	109	Black (4), Blue (19), Brown (10), Green (1), Lightcyan(1), Lightyellow(1), Magenta (3), Pink (1), Red (11), salmon(1), Turquoise (43), Yellow (14).	glioma [47]	Known	-	Unknown
6	miR-519a	12	109	Black (4), Blue (19), Brown (10), Green (1), Lightcyan(1), Lightyellow(1), Magenta (3), Pink (1), Red (11), salmon(1), Turquoise (43), Yellow (14).	down syndrome related AD [77]	Known	-	Unknown
7	miR-519c-3p	12	109	Black (4), Blue (19), Brown (10), Green (1), Lightcyan(1), Lightyellow(1), Magenta (3), Pink (1), Red (11), salmon(1), Turquoise (43), Yellow (14).	neuroblastoma [77]	Known	-	Unknown
8	miR-495	12	102	Black (3), Blue (17), Brown (8), Green (7), Greenyellow(1), Lightcyan(1), Lightyellow(1), Magenta (2), purple(2), Red (8), Turquoise (40), Yellow (12).	HIV mediated dementia [39]	Known	Age mediated skeletal muscle contamination [54]	Known
9	miR-944	11	138	Black (6), Blue (22), Brown (18), Green (3), Lightyellow(1), Magenta (1), Midnightblue(1), Purple(1), Red (11), Turquoise (57), Yellow (17).	Cancer metastasis in brain [70]	Known	-	Unknown
10	miR-664	11	123	Black (10), Blue (17), Brown (9), Cyan(1), Green (6), Magenta (2), Purple(1), Red (13), Tan(1) Turquoise (46), Yellow (17).	Psychiatric and Neurodegenerative disease [44].	Known	-	Unknown

Table 7 shows the detected inter-species multi-stage co-methylated modules (i.e., Black, Blue, Brown, Cyan, Green, Greenyellow, Grey60, Lightcyan, Lightyellow, Magenta, Midnightblue, Pink, Purple, Red, Salmon, Tan, Turquoise and Yellow). The number of containing genes in these eighteen modules are 65, 218, 167, 1, 63, 4, 2, 2, 3, 16, 2, 4, 9, 89, 1, 2, 451 and 2 genes, respectively. In case of Black module, 66 miRNAs target different genes, whereas 25 TFs regulate different genes. For this module, we identify two important KEGG pathways Viz., KEGG pathway of Alzheimer’s disease (*p*-value = 8.41E-13) and Neuro-Trophin Signaling pathway (*p*-value = 5.46E-6). Several gene-ontology terms are also associated with different genes of the Black module viz., GO:CC of Postsynaptic density (*p*-value = 2.3E-2); GO:MFs of Zinc ion binding (*p*-value = 1.7E-3), and cytokine binding (*p*-value = 0.00895). For Blue module, 83 miRNAs target different genes from this module, whereas 71 TFs regulate different genes from this module. For this module, we identify two important KEGG pathways Viz., Tight junction (*p*-value = 1.1E-2), and phatidylinositol signaling system (*p*-value = 2.0E-2). Like the previous module, different genes have been associated with several gene ontology terms Viz., GO:BPs of Intracellular signaling transduction (*p*-value = 4.2E-4), and cell morphogenesis (*p*-value = 2.7E-3); GO:CCs of Synaptosome (*p*-value = 4.7E-3), and histone methyl transferase complex (*p*-value = 2.9E-2). For Brown module, 53 miRNAs target different genes of the module, whereas 59 TFs regulate different genes of the module. For this module, the related significant gene-ontology terms for the different genes of the Brown module are GO:BPs of Neuron development (*p*-value = 2.2E-2), and cell morphogenesis (*p*-value = 2.6E-2); GO:CC of intracellular organelle lumen (*p*-value = 0.019554); GO:MFs of Protein kinase activity (*p*-value = 1.82E-8), Protein serine/threonine kinase activity (*p*-value = 4.33E-07), MAP kinase tyrosine/serine/threonine phosphatase activity (*p*-value = 0.004947), Protein tyrosine kinase activity (*p*-value = 0.012018). For Cyan module, 5 miRNAs target different genes of this module, whereas 4 TFs regulate different genes of this module. In case of Green module, 27 miRNAs target different genes of the module, whereas 9 TFs regulate different genes from this module. We identify gene-ontology terms that are associated with the different genes of this module Viz., GO:CCs of plasma membrane part (*p*-value = 2.8E-2), and golgi apparatus part (*p*-value = 4.4E-2). For Greenyellow module, 6 miRNAs target different genes of the module, whereas 4 TFs regulate different genes of the module. Grey60 module is connected with 17 targeter miRNAs and 3 regulator TFs. However, 14 miRNAs target different genes of Lightcyan module, whereas one TF regulates different genes of the Lightcyan module. The associated gene-ontology terms with this module are GO:BPs of branching morphogenesis of an epithelial tube (*p*-value = 4.8E-3), and angiogenesis (*p*-value = 1.1E-2). For Lightyellow module, 26 miRNAs target different genes of the module, whereas one TF regulates different genes of the module. In this case, two important KEGG pathways are identified i.e., B-cell receptor signaling pathway (*p*-value = 2.9E-2), and T-cell receptor signaling pathway (*p*-value = 4.2E-2). Different genes of Lightyellow associate with some gene-ontology terms viz., GO:BPs of negative regulation of transport (*p*-value = 2.0E-2), and regulation of cellular localization (*p*-value = 3.6E-2). Different genes of Magenta module are targeted by 3 miRNAs and regulated by 6 TFs. The related GO:CCs for this module is synapse (*p*-value = 3.6E-2). Thereafter, one miRNA targets different genes of Midnightblue module, whereas one TF regulates different genes of the Midnightblue module. In case of Pink module, three miRNAs target different genes of the pink module. We have identified related GO:BP of response to organic cycle substance (*p*-value = 1.8E-2). In case of Purple module, five TFs regulate different genes of the module. In case of Red module, 80 miRNAs target different genes of the module, whereas 11 TFs regulate different genes of the module. For Salmon module, we have identified 44 miRNAs targeting different genes of this module and two TFs regulating different genes of this module. After that, 250 miRNAs target different genes of Turquoise module, whereas 130 TFs regulate different genes of that module. Here, we find one significant related KEGG pathway i.e., adherens junction (*p*-value = 3.0E-2). The related significant gene-ontology terms associated with this module are GO:BPs of negative regulation of signal transduction (*p*-value = 7.7E-3), negative regulation of cell communication (*p*-value = 1.1E-2); GO:CC of Neuron projection (*p*-value = 3.4E-2); GO:MFs of protein complex binding (*p*-value = 6.3E-3), SMAD binding (*p*-value = 1.3E-2), DNA binding (*p*-value = 4.5E-2), enzyme binding (*p*-value = 4.6E-2), and ion binding (*p*-value = 4.9E-2). Finally, 62 miRNAs target different genes of Yellow module, whereas 82 TFs are identified that regulate different genes of the Yellow module. We get an important KEGG pathway i.e., MAPK signaling pathway (*p*-value = 1.5E-2). Besides this pathway, several gene-ontology terms are also associated with different genes of the Yellow module. These are GO:BPs of hindbrain morphogenesis (*p*-value = 2.3E-2), hindbrain development (*p*-value = 2.4E-2), and Ras protein signal transduction (*p*-value = 2.3E-2); GO:CCs of Cell junction (*p*-value = 4.9E-5), Synapse (*p*-value = 2.4E-2), Postsynaptic density (*p*-value = 3.3E-2); GO:MFs of GTPase regulator activity (*p*-value = 2.1E-5), transcription factor binding (*p*-value = 4.3E-2).

Table 8 provides the list of top ten TFs for the inter-species study. Among the top ten such TFs, two TFs (viz., **ZNF771** and **MYST3**) are novel (unknown) in both human as well as rhesus; whereas four (viz., ZNF286A, E2F4, SP1 and ZBTB20) are known (i.e., known to both human and rhesus), and remaining four TFs (viz., ZNF423, SP3, LBX1 and FOXM1) are partially known (i.e., known to only human, but unknown to rhesus). Notably, according to [64], ZNF286A has a connection with the identification of “plasticity induced late response genes” in human; and as mentioned in [65], ZNF286A involves in Hemi-parkinson’s disease in rhesus. Besides that, E2F4 is associated with Neurodegeneration disorder [32] in human body and SIV encephalitis [45] in rhesus body; whereas SP1 is linked in “Disrupting in early Huntington’s disease” [63] in human, and “mediate members of brain aging proteins and enhancing the chances of Alzeimer’s Disease” [66] in rhesus. Besides that, ZNF423 is responsible for Joubart’s Syndrome [67] in human, but no link between ZNF423 and rhesus brain related disease has been found in literature. ZBTB20 is involved in Age associated brain disease [46] in rhesus whereas ZBTB20 is associated with major depressive disorder [68] in case of human. SP3 is liable for Neurodegeneration disease [69] in human body, but in rhesus body, there is no association between SP3 and brain related disease. FOXM1 is responsible for Glioma [47] in human, but for rhesus body, no connection is recognized between FOXM1 and brain related disease. From Table 8, ZNF286A, E2F4, ZNF771, ZNF423, SP1, ZBTB20, SP3, LBX1, MYST3 and FOXM1 regulate 31, 61, 46, 42, 41, 42, 29, 28, 24 and 19 genes, respectively. Among the 31 genes regulated by ZNF286A, 7 genes belong to turquoise module, another 7 exist in yellow module, whereas remaining 1, 2, 3, 1, 6, 1, 1 and 2 genes are associated with black, blue, brown, cyan, green, magenta, purple and red modules, respectively. Among the 61 genes regulated by E2F4, the number of regulated genes in Black, Blue, Brown, Green, Magenta, Midnightblue, Pink, Turquoise and Yellow modules are 3, 14, 13, 4, 1, 1, 2, 15 and 8, respectively. The detailed descriptions regarding these are given in Table 8.

Similarly, Table 9 shows the top ten miRNA-list for the inter-species study. Among the top ten miRNAs, every miRNA is known in human related different pathways. Following that, miR-19b, miR-19a, miR-520d-5p, miR-524-5p, miR-519b-5p, miR-519a, miR-519c-3p, miR-495, miR-944 and miR-664 regulate 121, 119, 130, 130, 109, 109, 109, 102, 138, 123 genes, respectively. Although hsa-miR-944 is described as an important material for cancer metastasis in brain in [70], there are very few supporting documented research results to verify this point. Two miRNAs (viz., miR-524-5p and miR-495) are known (i.e., known to both human and rhesus), and remaining eight miRs (viz., miR-19b, miR-19a, miR-520d-5p, miR-519b-5p, miR-519a, miR-519c-3p, miR-944 and miR-664) are partially known (i.e., known to only human, but still unknown to rhesus). Here, two miRNAs are associated with Alzheimer’s Disease i.e., hsa-miR-19b and hsa-miR-520d-5p. In [71], hsa-miR-19b-3p is associated with Alzheimer’s Disease (hsa-miR-19b-3p is reducing number during Alzheimer’s Disease), but miR-19b is still unknown in rhesus. Notably, hsa-miR-19b-3p is connected with fourteen different gene-modules (viz., black, blue, brown, green, grey60, lightyellow, magenta, pink, purple, red, salmon, tan, turquoise and yellow). From these fourteen modules, collectively one hundred twenty one genes are targeted. Turquoise contains highest number of genes (i.e., forty five genes) which are targeted by miR-19b. Next, yellow module has twenty one genes targeted by miR-19b. However, two HIV mediated diseases are pointed here namely, HIV mediated Neurodegenrative diseases [39] and HIV mediated dementia [39] that are created by two different miRs viz., hsa-miR-19a and hsa-miR-495, respectively. The detailed description regarding these are demonstrated in Table 9.

In terms of intra species analysis, number of color modules is lower than inter species analysis. However, number of modules in human is higher than rhesus. It is proper evidence to comment on methylation level variety of rhesus and human. On other hand, in mammals brain tissue specific methylation study gives a proper insight on differential methylation and diseases. The brain specific diseases, incorporated with unusual methylation level and hydroxy methylation level, has already been mentioned. The effect of miRNA degradation influences methylation and hydroxymethylation for cancers. From the study, it can be observed that brain related diseases are associated with miRNA and methylation. There is a chance to observe similar type of functionality in case of brain related diseases. So from these extensive study, an insight to therapeutic aspects can be discussed over the whole disease signaling pathway depending on controlling unusual methylation and hydroxy methylation rate.

## Conclusion

In this article, we provide a novel study (viz, intra and inter-species study) between human and rhesus on the brain tissue of 5mC and 5hmC data-profiles through TF-miRNA-gene network based gene module identification. The gene-modules are the verified by KEGG pathway and gene-ontology is analyzed by DAVID database on the gene representing the individual module. Moreover, top ten regulator TFs and targeter miRNAs that connect the maximum number of gene-modules for the intra-species study as well as the inter-species analysis, are determined. During intra-species analysis, for human, two regulator TFs (viz., MYST3 and ZNF771) whereas three regulator for rhesus TFs (viz., BAZ2B, RCOR3 and ATF1), and two targeter miRNAs (namely, mml-miR-768-3p and mml-miR-561) are identified novel. For the inter-species study, we have identified two novel TFs (i.e., ZNF771 and MYST3) of which the related information regarding both human and rhesus are unknown in literature. These TFs/miRNAs might be highly responsible for any brain related disease. We have highlighted the participating genes of each intra-species and the inter-species module along with the regulator TFs or targeter miRNAs that are connected with several brain-related dreadful diseases as well as rare neglected diseases (viz., wolf Hirschhorn syndrome, Joubart’s Syndrome, Huntington’s disease, SIV mediated enchaphilits, Parkinson’s Disease, Bipolar disorder and Schizophenia etc.). For example, a TF namely ZNF423 is connected with Joubart’s Syndrome in human, and TF namely SP1 is associated with “Disrupt in early Huntington’s disease” in human, whereas hsa-miR-34c-5p is responsible for Bipolar disorder and Schizophenia in human, and miR-524-5p is associated with Amyotrophic lateral sclerosis in human. From the rate of diversity in gene methylation profiling, the variety in tissue specific gene samples can be understood. In terms of number of color modules, rate of diversity in human is greater than that in rhesus. Finally, as discussed in resulting section, there is chance to derive some therapeutic aspects by studying tissue specific signaling pathways at epigenetic level.

## Additional files

Additional file 1 Complete list of MicroRNA targeters and TF regulators of color modules from Human Intra-Species analysis. A table of all MicroRNA targeters and TF regulators of color modules from Human Intra-species analysis. (XLSX 32 kb)

Additional file 2 Color modules for rhesus and corresponding TF regulator and MicroRNA targeter where 5hmC is controlled and 5mC is diseased. A table of generated color modules for rhesus samples where 5hmC is controlled and 5mC is diseased and corresponding predicted TF regulators and miRNAs are also mentioned. (XLSX 12 kb)

Additional file 3 Color modules for Human and corresponding TF regulator and MicroRNA targeter where 5hmC is controlledand 5mC is diseased. A table for Generated color modules for human samples where 5hmC is controlled and 5mC is diseased and corresponding predicted TF regulators and miRNAs are also mentioned. (XLSX 13 kb)

Additional file 4 Complete list of MicroRNA targeters and TF regulators of color modules from Rhesus monkey Intra-Species analysis. A table of all MicroRNA targeters and TF regulators of color modules from Rhesus Intra-species analysis. (XLSX 28 kb)

## Abbreviations

5mC: 5-methylcytosine
5hmC: 5-hydroxymethylcytosine
DNMTs: DNA methyltransferase
TFs: Transcription factors
MiRs: MicroRNAs
KEGG: Kyoto Encyclopedia of Genes and Genomes
